# Lymphocytic colitis in the course of a treatment with sertraline. A case report

**DOI:** 10.1192/j.eurpsy.2022.1845

**Published:** 2022-09-01

**Authors:** A. Cerame, A. Cotillas, P. Coucheiro, A. Franco Soler

**Affiliations:** 1 Hospital Universitario José Germain, Hospital De Día, Leganes, Spain; 2 Centro de Salud San Blas, Family Medicine, Parla, Spain

**Keywords:** lymphocytic colitis, side-effect, sertraline

## Abstract

**Introduction:**

Lymphocytic colitis is an unusual side-effect of the treatment with SSRIs particularly sertraline. According to several sources, this side effect could be underdiagnosed and underreported.

**Objectives:**

We present the case of a 31-year-old female patient who presented watery non-bloody diarrhea for a month. The patient had been diagnosed with Persistent Depressive Disorder six years prior to the episode and was being treated with sertraline.

**Methods:**

A case report is presented alongside a review of the relevant literature regarding Lymphocytic colitis, its differential diagnosis and treatment.

**Results:**

The case was coordinated between the out-patient psychiatry department and the patient’s Family Medicine specialist. The patient underwent a complete panel of tests including blood and stool test, and colonoscopy. The biopsy showed results compatible with the aforementioned diagnosis. Since the only pharmacological treatment which could cause or trigger lymphocytic colitis was sertraline, it was removed and treatment with oral budesonide was started with good results.

**Conclusions:**

Lymphocytic colitis in the context of the treatment with SSRIs, particularly sertraline, is a side-effect which is more prevalent than what was thought in the past. A close coordination between psychiatrists and GP is of vital importance for the adequate treatment of mental health problems and treatment’s side-effects. It is important to bear in mind this possible side-effect to increase patient’s safety.

**Disclosure:**

No significant relationships.

